# Factors Predicting Locoregional Recurrence After Neoadjuvant Chemotherapy and Nipple-Sparing/Skin-Sparing Mastectomy With Immediate Breast Reconstruction

**DOI:** 10.3389/fonc.2021.675955

**Published:** 2021-07-01

**Authors:** Zhen-Yu Wu, Hee Jeong Kim, Jong Won Lee, Il Yong Chung, Jisun Kim, Sae Byul Lee, Byung-Ho Son, Jin Sup Eom, Jae Ho Jeong, Gyungyub Gong, Hak Hee Kim, Sei-Hyun Ahn, BeomSeok Ko

**Affiliations:** ^1^ Department of Breast Surgery, Shanghai East Hospital, Tongji University School of Medicine, Shanghai, China; ^2^ Division of Breast Surgery, Department of Surgery, Asan Medical Center, University of Ulsan College of Medicine, Seoul, South Korea; ^3^ Department of Plastic Surgery, Asan Medical Center, University of Ulsan College of Medicine, Seoul, South Korea; ^4^ Department of Oncology, Asan Medical Center, University of Ulsan College of Medicine, Seoul, South Korea; ^5^ Department of Pathology, Asan Medical Center, University of Ulsan College of Medicine, Seoul, South Korea; ^6^ Department of Radiology, Asan Medical Center, University of Ulsan College of Medicine, Seoul, South Korea

**Keywords:** breast cancer, immediate breast reconstruction, skin-sparing mastectomy, nipple-sparing mastectomy, neoadjuvant chemotherapy, locoregional recurrence, risk factor

## Abstract

**Background:**

Few data are available on the risk factors of locoregional recurrence (LRR) after neoadjuvant chemotherapy (NACT) and immediate breast reconstruction (IBR) in breast cancer. Herein, we evaluated the factors predicting LRR in a large series of patients who underwent either nipple- (NSM) or skin-sparing mastectomy (SSM) with IBR after NACT.

**Methods:**

We retrospectively analyzed 609 breast cancer patients who underwent NACT and NSM/SSM with IBR between February 2010 and June 2017. Factors associated with an increased risk of LRR were analyzed by univariate (chi-square or Fisher’s exact test) and multivariate (Cox proportional hazard regression model) analyses.

**Results:**

During a median follow-up of 63 months, LRR as the first event occurred in 73 patients, and the 5-year cumulative LRR rate was 10.8%. Multivariate analysis revealed post-NACT Ki67 ≥ 10% [hazard ratio (HR), 2.208; 95% confidence interval (CI), 1.295-3.765; *P* = 0.004], high tumor grade (HR, 1.738; 95% CI, 1.038-2.908; *P* = 0.035), and presence of lymphovascular invasion (LVI) (HR, 1.725; 95% CI, 1.039-2.864; *P* = 0.035) as independently associated with increased LRR risk. The 10-year LRR rate was 8.5% for patients with none of the three associated risk factors, 11.6% with one factor, 25.1% with two factors, and 33.7% with all three factors (*P* < 0.001).

**Conclusions:**

Post-NACT Ki67 ≥ 10%, high tumor grade, and presence of LVI are independently associated with an increased risk of developing LRR after NACT and NSM/SSM with IBR. Future prospective trials are warranted to decrease the risk of LRR in patients with associated risk factors.

## Introduction

Neoadjuvant chemotherapy (NACT) has been established as the standard of care for locally advanced breast cancer and is now being used more often as a treatment in early-stage breast cancer (1). NACT aims to increase the rate of breast conservation; however, a large proportion of patients receiving NACT undergo mastectomy as the surgical treatment, either because breast-conserving surgery is not feasible or because of patient preference. Over the last decade, patients have begun to prefer nipple- (NSM) or skin-sparing mastectomy (SSM) combined with immediate breast reconstruction (IBR) in the treatment of breast cancer, as it provides improved aesthetic results and quality of life (2, 3). Several non-randomized studies have demonstrated that the oncologic outcomes of NSM/SSM with IBR are comparable to those of conventional mastectomy alone (4–6). Recently, NSM/SSM with IBR has also been performed in patients who receive NACT; however, data related to the long-term safety of such treatments in this patient population are still insufficient (7). In addition, locoregional recurrence (LRR) following NSM/SSM with IBR remains clinically challenging, not only because it may indicate poor prognosis (8), but also because the oncologic management of LRR may lead to loss of the initial reconstruction (9). In patients who receive NACT and breast reconstruction, the predictive value of clinicopathologic features or treatment-associated factors for LRR is unclear due to a lack of data.

In this study, we aimed to identify the factors associated with an increased risk of LRR in a large series of breast cancer patients who underwent NSM/SSM with IBR after NACT.

## Materials and Methods

This study was approved by the institutional review board (IRB) of Asan Medical Center, Seoul, Republic of Korea (No. 2017-1341). This study is a retrospective study conducted with the exemption of consent under IRB deliberation using a platform for extracting unidentified clinical information for research purposes. The medical records of all patients who underwent IBR with NSM/SSM after NACT for primary breast cancer between January 2010 and June 2017 at the Asan Medical Center, Seoul, Republic of Korea, were reviewed from a prospectively maintained database. Patients presenting with inflammatory breast cancer or synchronous distant metastasis were excluded. Patient and tumor characteristics were collected and analyzed, including age at diagnosis, tumor stage, grade, molecular subtype, histotype, lymphovascular invasion (LVI) status, presence of extensive intraductal component, post-NACT Ki67 status, and pathological multifocality/multicentricity. Tumor staging was conducted according to the 8th American Joint Committee on Cancer Staging Manual (10). Pathological complete response (pCR) was defined as no evidence of invasive cancer in the breast or axillary lymph nodes.

All patients included in this study received NACT after breast cancer diagnosis. The NACT regimens were selected at the discretion of the treating oncologist. NSM/SSM was performed by breast surgeons, and IBR was performed by plastic surgeons using autologous flaps or implants. NSM or SSM was performed according to the indications of conventional mastectomy, regardless of tumor size or tumor-to-nipple distance, as long as there was no evidence of tumor involvement in the breast skin and nipple-areola complex, clinically or on imaging. In cases of NSM, retroareolar frozen-section biopsy specimens were collected and examined intraoperatively. The nipple-areola complex was preserved if the shape, color, and palpated features of the nipple were normal, and if the nipple margin was confirmed to be tumor free on frozen-section biopsy. In cases in which the retroareolar tissue was positive for malignancy in the frozen section or permanent biopsy, the nipple with or without the areola was removed, and these cases were considered SSM. The decision to undergo adjuvant radiotherapy was made by the treating radiation oncologist after consideration of pre- and post-NACT disease stages, tumor response to NACT, and other tumor biomarkers in patients. Most patients who required adjuvant radiotherapy after evaluation underwent simultaneous irradiation of the chest wall and supraclavicular region. Adjuvant hormonal therapy was applied in patients with hormone receptor-positive disease.

Postoperatively, patients were regularly followed up every 3–6 months for the first 5 years and annually thereafter. Recurrence and metastasis were identified based on the results of the clinical examination, chest radiography, and tumor marker (CA15–3) measurements, which were taken every follow-up visit. In some cases, abnormal clinical findings were further evaluated using chest computed tomography (CT), a bone scan, ultrasonography, and/or positron emission tomography-CT. In patients suspected of LRR, fine needle aspiration, core needle, or excisional biopsy was performed for pathological confirmation. Lesions with clear evidence of distant metastasis on imaging evaluation were considered as recurrence without pathological examination.

LRRs were classified as local or regional recurrence. Local recurrence was defined as biopsy-proven recurrences in the ipsilateral skin/subcutaneous layer, chest wall, or nipple-areola complex, and regional recurrence was defined as carcinoma metastases in the ipsilateral axillary, supraclavicular, or internal mammary lymph node. Any other site of recurrence was considered distant metastasis. Patients with initial distant metastasis were excluded from the LRR group. In cases of concurrent LRR and distant metastasis, each recurrence was counted as an event. Occurrence of contralateral breast cancer was considered a new primary cancer and was not counted as a recurrence. Follow-up was calculated from the date of diagnosis.

The 5- and 10-year cumulative LRR rates were calculated using the Kaplan-Meier method and compared using the log-rank test between subgroups. The clinicopathological factors that were significant in univariate analyses (Chi-square or Fisher’s exact test) of LRR were included in the multivariate analysis using the Cox proportional hazards regression model. All statistical analyses were performed using IBM SPSS Statistics software version 24.0 for Windows (IBM Corp., Armonk, NY, USA). Two-tailed *P*-values < 0.05 were considered significant.

## Results

A total of 609 patients who underwent NACT and IBR with NSM/SSM for primary breast cancer were included. Patient, tumor, and treatment characteristics are shown in **Table 1**.

**Table 1:** Patient, tumor, and treatment characteristics (N=609).

Characteristics		N	%
Age at diagnosis, years	Median	42 (23–72)	
	≤40	254	41.7
	>40	355	58.3
Clinical T stage	cT1	38	6.2
	cT2	355	58.3
	cT3-4	216	35.5
Clinical N stage	cN0	212	34.8
	cN1	313	51.4
	cN2-3	84	13.8
Pathological T stage	ypT0/ypTis	87	14.3
	ypT1	217	35.6
	ypT2	220	36.1
	ypT3	85	14.0
Pathological N stage	ypN0	287	47.1
	ypN1	221	36.3
	ypN2-3	101	16.6
Molecular subtype	HR+/HER2-	323	53.0
	HR+/HER2+	159	26.1
	HR-/HER2+	64	10.5
	TN	63	10.3
pCR	Yes	79	13.0
	No	530	87.0
Pathological MF/MC	Yes	206	33.8
	No	403	66.2
Histotype	Ductal	533	87.5
	Lobular	26	4.3
	Mixed/Others	50	8.2
Tumor grade	1	15	2.5
	2	436	71.6
	3	158	25.9
LVI	Yes	227	37.3
	No	382	62.7
Extensive intraductal component	Yes	170	27.9
	No	439	72.1
Post-NACT Ki67	<10%	281	46.1
	≥10%	255	41.9
	Unknown	73	12.0
NACT regimens	AC/AC+T	546	89.7
	T	51	8.4
	Others	12	2.0
Mastectomy type	NSM	370	60.8
	SSM	239	39.2
Axillary surgery	SLNB alone	359	58.9
	ALND	250	41.1
Adjuvant radiotherapy	Yes	316	51.9
	No	293	48.1
Adjuvant hormonal therapy	Yes	482	79.1
	No	127	20.9
Adjuvant chemotherapy	Yes	70	11.5
	No	539	88.5
Trastuzumab in HER2+	Yes	219	98.2
	No	4	1.8
Reconstructive surgery	Autologous flaps	420	69.0
	Implants	189	31.0

AC, anthracycline; ALND, axillary lymph node dissection; HER2, human epidermal growth factor receptor 2; HR, hormone receptor; LVI, lymphovascular invasion; MF/MC, multifocality/multicentricity; NACT, neoadjuvant chemotherapy; NSM, nipple-sparing mastectomy; pCR, pathological complete response; SLNB, sentinel lymph node biopsy; SSM, skin-sparing mastectomy; T, taxane; TN, triple negative.

The median age at diagnosis was 42 years (range, 23-72 years). The majority (89.7%) of patients received anthracycline-based (with or without taxane) NACT. NSM was performed in 370 (60.8%) patients and SSM in 239 (39.2%). Four hundred and twenty (69%) patients underwent autologous flap reconstruction, and 189 (31%) patients underwent implant-based reconstruction. Adjuvant radiotherapy was administrated in 316 (51.9%) patients. Among the 223 patients with human epidermal growth factor receptor 2 (HER2)-positive disease, 219 (98.2%) received adjuvant trastuzumab. On follow-up, pCR was observed in 79 (13%) patients.

The median follow-up period was 63 months (range, 11-135 months). LRR as the first event occurred in 73 patients, and the 5-year cumulative LRR rate was 10.8%. Among these, isolated LRR occurred in 55 patients (75.3%) and concurrent LRR with distant metastasis occurred in 18 (24.7%). **Table 2** summarizes the oncologic outcomes of the entire cohort. The median time to LRR was 35 months (range, 7-76 months). Patients with isolated LRR as the first event showed a significantly lower 10-year overall survival rate than those without LRR (64.7% *vs*. 90.2%; log-rank *P* = 0.035). **Table 3** shows the incidence rates of LRR according to various clinicopathological and treatment factors. The following factors were significantly associated with increased rates of LRR in the univariate analysis: age at diagnosis ≤ 40 years, pathological T stage, pathological nodal status, pCR status, tumor grade, LVI, and post-NACT Ki67 status. Of these, post-NACT Ki67 ≥ 10% [hazard ratio (HR), 2.208; 95% confidence interval (CI), 1.295-3.765; *P* = 0.004], high tumor grade (HR, 1.738; 95% CI, 1.038-2.908; *P* = 0.035), and presence of LVI (HR, 1.725; 95% CI, 1.039-2.864; *P* = 0.035) were independently associated with reduced LRR-free survival in the multivariate analysis (**Table 4**).

**Table 2:** Oncologic outcomes.

	N	%
Locoregional recurrence	73^a^	12
Skin/chest wall	27	4.4
Nipple-areola complex	7	1.9^b^
Regional lymph nodes	45	7.4
Distant metastasis	99	16.3
Any first recurrence	138	22.7
Death	57	9.4
5-y locoregional recurrence-free survival		87.6
5-y disease-free survival		77.5
5-y distant metastasis-free survival		83.6
5-y overall survival		92.3

a Including 5 cases of concurrent local and regional recurrence without distant metastasis, and 18 cases of concurrent local and/or regional recurrence with distant metastasis as the first event.

b Calculated in 370 cases of nipple-sparing mastectomy.

**Table 3:** Univariate analysis of factors associated with LRR.

Variables		N	LRR rate, %	*P*-value
		73	12.0	
Age at diagnosis, years	≤40	39	15.4	0.030
	>40	34	9.6	
Clinical T stage	cT1	4	10.5	0.530
	cT2	47	13.2	
	cT3-4	22	10.2	
Clinical N stage	cN0	22	10.4	0.669
	cN1	40	12.8	
	cN2-3	11	13.1	
Pathological T stage	ypT0/ypTis	4	4.6	0.045
	ypT1	33	15.2	
	ypT2	29	13.2	
	ypT3	7	8.2	
Pathological nodal status	ypN-	25	8.7	0.019
	ypN+	48	14.9	
Molecular subtype	HR+/HER2-	35	10.8	0.362
	HR+/HER2+	17	10.7	
	HR-/HER2+	11	17.2	
	TN	10	15.9	
pCR	Yes	3	3.8	0.015
	No	70	13.2	
Pathological MF/MC	Yes	30	14.6	0.162
	No	43	10.7	
Tumor grade	1, 2	45	10.0	0.010
	3	28	17.7	
LVI	Yes	40	17.6	0.001
	No	33	8.6	
Extensive intraductal component	Yes	22	12.9	0.652
	No	51	11.6	
Post-NACT Ki67	<10%	24	8.5	0.001
	≥10%	47	18.4	
	Unknown	2	NA	
Mastectomy type	NSM	46	12.4	0.674
	SSM	27	11.3	
Axillary surgery	SLNB alone	37	10.3	0.126
	ALND	36	14.4	
Adjuvant radiotherapy	Yes	31	9.8	0.086
	No	42	14.3	
Adjuvant hormonal therapy	Yes	52	10.8	0.076
	No	21	16.5	
Adjuvant chemotherapy	Yes	10	14.3	0.529
	No	63	11.7	
Trastuzumab in HER2+	Yes	28	12.6	1.000
	No	0	0.0	
Reconstructive surgery	Autologous flaps	50	11.9	0.926
	Implants	23	12.2	

ALND, axillary lymph node dissection; HER2, human epidermal growth factor receptor 2; HR, hormone receptor; LRR, locoregional recurrence; LVI, lymphovascular invasion; MF/MC, multifocality/multicentricity; NA, not applicable; NACT, neoadjuvant chemotherapy; NSM, nipple-sparing mastectomy; pCR, pathological complete response; SLNB, sentinel lymph node biopsy; SSM, skin-sparing mastectomy; TN, triple negative.

**Table 4:** Multivariate analysis of risk factors associated with LRR.

Variables		HR	95% CI	*P*-value
Age at diagnosis, years	>40	1 (reference)		0.427
	≤40	1.214	0.752-1.959	
Pathological T stage	ypT0/ypTis	1 (reference)		
	ypT1	1.888	0.367-9.708	0.447
	ypT2	2.017	0.867-4.692	0.103
	ypT3	1.857	0.806-4.278	0.146
Pathological nodal status	ypN-	1 (reference)		0.097
	ypN+	1.589	0.920-2.745	
pCR	Yes	1 (reference)		0.452
	No	2.971	0.174-50.602	
Tumor grade	1, 2	1 (reference)		0.035
	3	1.738	1.038-2.908	
LVI	No	1 (reference)		0.035
	Yes	1.725	1.039-2.864	
Post-NACT Ki67	<10%	1 (reference)		0.004
	≥10%	2.208	1.295-3.765	

CI, confidence interval; HR, hazard ratio; LRR, locoregional recurrence; LVI, lymphovascular invasion; NACT, neoadjuvant chemotherapy; pCR, pathological complete response.

**Figure 1:**
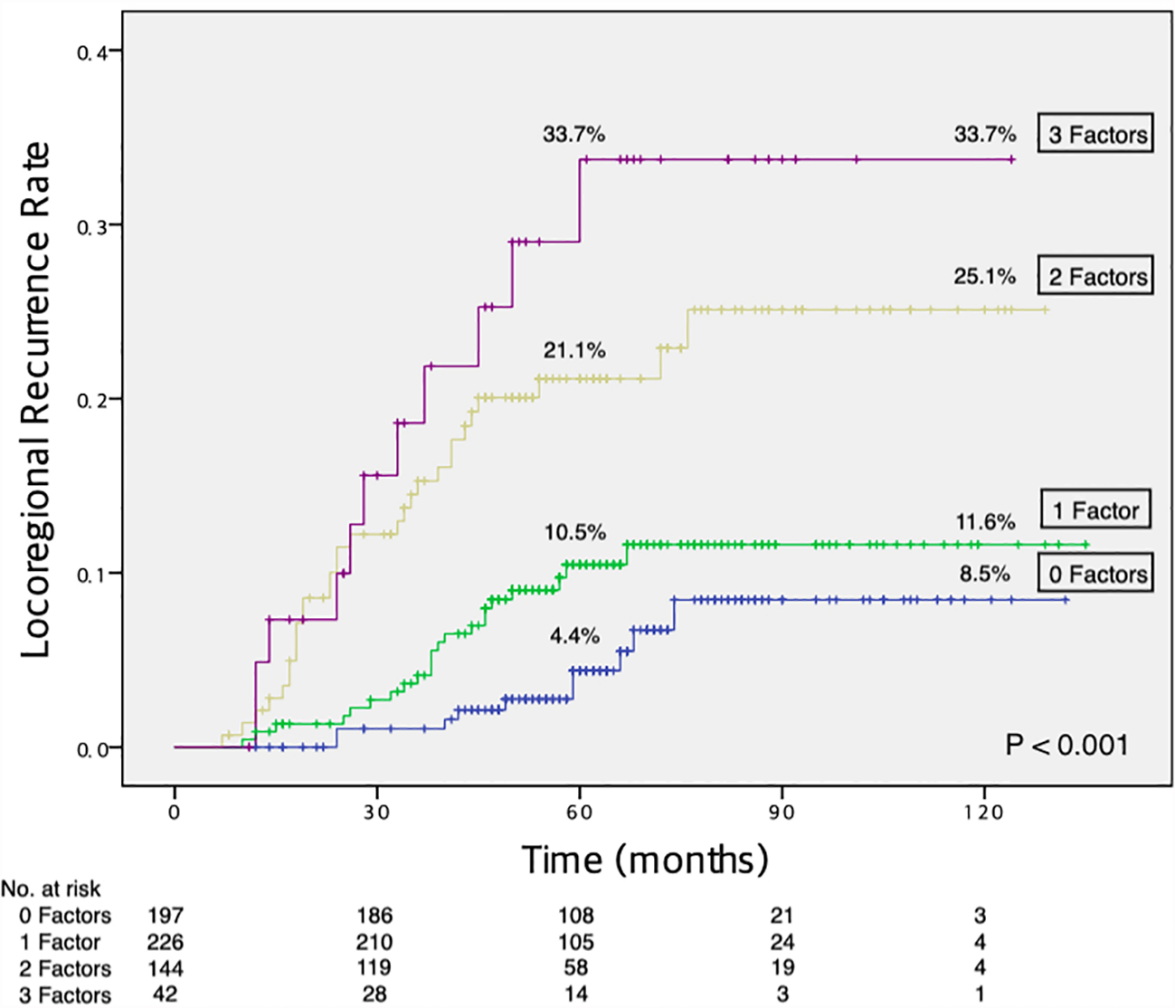
Increased risk of LRR with an increasing number of independent risk factors. LRR, locoregional recurrence.

**Figure 1** shows the Kaplan-Meier curves for LRR risk, according to the number of independent risk factors. The 10-year rate of LRR was 8.5% for patients with none of the three independent risk factors (n = 197, 32.3%), 11.6% for those with one risk factor (n = 226, 37.1%), 25.1% for those with two risk factors (n = 144, 23.6%), and 33.7% for those with all three risk factors (n = 42, 6.9%; log-rank *P* < 0.001).

## Discussion

Although previous studies have investigated predictive factors of LRR after NACT in conventional mastectomy or breast-conserving surgery (11–15), little data regarding the risk factors of LRR after NACT for NSM/SSM with IBR exists. In this study, we identified the 5-year LRR rate (10.8%) and factors predicting LRR in breast cancer patients who underwent NSM/SSM with IBR after receiving NACT. Post-NACT Ki67 ≥ 10%, high tumor grade, and presence of LVI were independent risk factors for LRR in the current setting. Notably, the 10-year LRR rate reached 33.7% in patients with all three risk factors and was 8.5% in patients with none of these factors.

NSM/SSM with IBR has become an important surgical strategy in modern breast cancer care. This surgical procedure, particularly NSM with IBR, can provide significantly improved aesthetic results, patient satisfaction, and/or psychosocial/sexual well-being (2, 3, 16). A recent analysis from the National Cancer Database of the American College of Surgeons and the American Cancer Society showed an increasing trend toward the application of NSM in patients with advanced disease, particularly in those who received NACT, and highlighted the importance of further prospective trials to validate the evidence of oncologic safety of this procedure (7). The current National Comprehensive Cancer Network (NCCN) guidelines recommend that NSM/SSM should be performed by an experienced breast surgery team working in a multidisciplinary fashion, according to specific clinical features and selected criteria (17). In case of NSM, NCCN guidelines include some cases of locally advanced invasive breast cancers, provided there is complete clinical response after NACT and no nipple involvement. Furthermore, assessment of nipple margin during surgery is mandatory (17). Several studies have reported on the feasibility of this approach in patients who receive NACT, and the LRR rates ranged between 3.2% and 10.3% (18–22). However, the majority of the studies involved a relatively small sample size and short follow-up durations. In the current study, with a median follow-up of 63 months, we found a 5-year cumulative LRR rate of 10.8% for the entire cohort. The LRR rate of our cohort appears acceptable in consideration of the previously reported LRR rates, which ranged from 6.0% to 21.0% after NACT and mastectomy with or without reconstruction (14, 23–26).

The occurrence of breast cancer LRR is an important determinant of adverse survival outcomes (8, 27–29). In our study, isolated LRR as the first event in patients who underwent NSM/SSM with IBR after NACT was associated with a poor 10-year overall survival rate. In addition, patients with isolated LRR often required oncologic management, including surgical excision of the recurrent tumor, which could result in loss of the initial reconstruction (9). Therefore, identifying risk factors for LRR in the current setting is imperative for optimal locoregional management and patient surveillance strategies. However, investigating risk factors for recurrence after NACT remains a challenge because of the high frequency of inconsistent disease status in patients between before and after neoadjuvant treatment. Previous studies have described several clinical and pathological factors of LRR after NACT. The National Surgical Adjuvant Breast and Bowel Project (NSABP) study, including NSABP B-18 and NSABP B-27 data, identified that young age (< 50 years), clinical tumor size (> 5 cm), clinical node status (cN+), and pCR status (ypT+ or ypN+) were predictive of an increased risk of LRR after NACT in patients who underwent mastectomy and breast conservation therapy (11). The authors developed a nomogram using these factors to predict the risk of LRR and guide optimal administration of adjuvant radiotherapy (11); however, histopathological characteristics such as molecular subtype, tumor grade, LVI, and Ki67 index were not analyzed in that study (11). One study by the European Organization for Research and Treatment of Cancer 10994/BIG 1-00 revealed that triple-negative or HER2-positive subtype and lack of pathologic response were associated with increased LRR after NACT (12). However, Ki67 index, tumor grade, and LVI were not analyzed in that study (12). Our current study investigated the risk factors of LRR exclusively in patients who underwent NSM/SSM with IBR after NACT and involved several prognostic factors not included in the aforementioned studies that used prospective data. Moreover, in our multivariate analysis, post-NACT Ki67 index, tumor grade, and LVI independently influenced LRR. In our univariate analysis, factors including age at diagnosis, pathological T stage, pathological node stage, and pCR status were associated with LRR rates; however, after multivariate analysis these factors were no longer significant. Notably, the role of post-NACT Ki67, tumor grade, and LVI in LRR risk has previously been suggested in smaller retrospective studies (13–15). In a study by Yamazaki et al., 217 patients who underwent NACT and breast-conserving surgery were analyzed, and post-NACT Ki67 > 20%, triple-negative subtype, the presence of LVI, and high tumor grade were found to be significant prognostic factors of LRR (13). However, these factors were identified in a univariate analysis, and no multivariate analysis was conducted (13). In another retrospective study by Wang et al. that included 217 patients with cT1-2N0-1 who underwent NACT and mastectomy, the 5-year LRR rate was 12%, and LVI, tumor grade, and ypN stage were independent prognostic factors of LRR in multivariate analysis (14). However, no data on the Ki67 index were presented in that study (14). In a previous retrospective study including 319 NSM cases after NACT conducted at our center demonstrated that post-NACT Ki67 index was the only independent risk factor for LRR in multivariate analysis (30). Our results on factors correlated with higher LRR risk after NACT are in line with those of previous reports (13–15, 30). In addition, we quantified LRR risk according to the number of independent risk factors and found that the 10-year LRR rate was 8.5% in patients with none of the three independent risk factors, while patients with one, two, or, three of these factors had 10-year LRR rates of 11.6%, 25.1%, and 33.7%, respectively. This risk stratification of LRR may aid in selecting patients who can benefit from further investigation of locoregional management (i.e., adjuvant radiotherapy) strategies in the current setting.

The current study was limited by its retrospective, single-center design, and the study population was heterogeneous for clinicopathological and treatment characteristics. Detailed analysis of the relationship between different adjuvant radiotherapy regimens and LRR, as well as the rate of reconstruction failure, could not be conducted in this study because relevant data were not available. In addition, a relatively small number of patients and LRR events were included in certain subgroups of interest, which might have affected the statistical power of the results.

In conclusion, post-NACT Ki67 ≥ 10%, high tumor grade, and presence of LVI are independently associated with a high risk of developing LRR after NACT and NSM/SSM with IBR. Future prospective trials are warranted to decrease the risk of LRR in patients with associated risk factors.

## Data Availability Statement

The original contributions presented in the study are included in the article/supplementary material. Further inquiries can be directed to the corresponding author.

## Ethics Statement

This study was approved by the institutional review board of Asan Medical Center, Seoul, Korea (No. 2017-1341). Written informed consent for participation was not required for this study in accordance with the national legislation and the institutional requirements.

## Author Contributions

Z-YW and BK: conception and design. S-HA and BK: administrative support. Z-YW, HK, JL, IC, JK, SL, B-HS, JE, JJ, GG, HK, and BK: data collection. Z-YW: data processing, analysis and manuscript writing. All authors contributed to the article and approved the submitted version.

## Conflict of Interest

The authors declare that the research was conducted in the absence of any commercial or financial relationships that could be construed as a potential conflict of interest.
